# Anxious‐depressive attack—A clinical state with intrusive rumination

**DOI:** 10.1002/pcn5.70396

**Published:** 2026-09-16

**Authors:** Hisanobu Kaiya, Takeshi Otowa, Tsukasa Sasaki, Shota Noda, Naomi Matsuda, Yojiro Sakai, Fusaka Minami

**Affiliations:** ^1^ Panic Disorder Research Center, Warakukai Medical Corporation Tokyo Japan; ^2^ Department of Psychiatry Kyoto Prefectural University of Medicine Kyoto Japan; ^3^ Akasaka Clinic, Warakukai Medical Corporation Tokyo Japan; ^4^ Department of Psychiatry Teikyo University School of Medicine Tokyo Japan; ^5^ Laboratory of Health Education, Graduate School of Education The University of Tokyo Tokyo Japan; ^6^ Yokohama Clinic, Warakukai Medical Corporation Yokohama Japan; ^7^ Department of Neuropsychiatry Keio University School of Medicine Tokyo Japan

**Keywords:** anxious‐depressive attack (ADA), maladaptive coping behaviors, reduced self‐reflective awareness, rejection sensitivity, self‐immersion

## Abstract

**Aim:**

The present study aimed to examine anxious‐depressive attack (ADA) regarding its characteristics and the circumstances of onset, thereby clarifying why ADA has remained underrecognized.

**Methods:**

In this cross‐sectional study, 286 psychiatric outpatients were assessed using structured clinical interviews. ADA was diagnosed according to predefined criteria, characterized by sudden and recurrent episodes beginning with intense anxious‐depressive distress, followed by prolonged intrusive rumination involving negative memories or future worries and prominent negative emotions. Data on prevalence, onset, temporal patterns, phenomenology, and coping behaviors were systematically collected.

**Results:**

The lifetime prevalence of ADA was 21.7%, with onset typically during adolescence or early adulthood. Episodes most commonly occurred during periods of mental disengagement, particularly in the evening or at night, and typically lasted 2–3 h. Detailed intrusive thoughts were predominantly autobiographical, emotionally intense, and often related to temporally distant experiences. While most patients could recall the onset, offset, and content of their episodes, 43.5% regarded ADA as a mental state that anyone might experience and 29.0% had never disclosed their experiences. Maladaptive coping behaviors, including suicidal behavior or sexual derailment, were observed in 40.3% of cases.

**Conclusion:**

ADA represents a distinctive mental state preserving consciousness and memory coexist with reduced self‐reflective awareness of the pathological nature of the episode. Its ego‐syntonic, covert nature, prolonged duration, and relative lack of prominent somatic symptoms may contribute to underrecognition of ADA. Recognizing ADA in clinical practice should facilitate more accurate clinical assessment suggesting an appropriate treatment strategy.

## INTRODUCTION

The authors initially identified anxious‐depressive attack (ADA) in patients with difficult‐to‐treat depression following remission from panic disorder (PD). Clinical observations revealed that ADA not only is not confined to PD but also occurs across a range of psychiatric conditions. ADA is characterized by the sudden onset of intense anxiety—described as a “flush of fear” (an abrupt surge of arousal)—accompanied by intrusive thoughts and profound feelings of anxiety or/and depression. These intrusive thoughts typically center on distressing past experiences or anticipated future concerns, which are repeatedly ruminated upon. This ruminative cycle leads to a sustained state of emotional distress and often gives rise to maladaptive coping behaviors.

Despite its clinical relevance, this constellation of symptoms is not currently recognized within standard psychiatric nosology. However, a similar phenomenology was described by Freud in his work on the justification for detaching a particular syndrome from neurasthenia,^1^ in which he proposed three phenotypic expressions of anxiety attacks: ① anxiety attacks consisting solely of emotional disturbance, ② anxiety attacks accompanied by negative ideation, and ③ anxiety attacks associated with somatic dysfunctions—now understood as panic attack (PA). The first type was named pure anxiety attack by us.^2^ The second type, characterized by ideational content, corresponds to what the present authors have termed “anxious‐depressive attack (ADA)” in previous reports.^3^, ^4^

Recently, the authors reported that the lifetime prevalence of ADA was 21.7% among new patients presenting to an anxiety disorder clinic. Furthermore, ADA was observed across 16 Axis I disorders and 6 Axis II personality disorders.^5^ The transdiagnostic nature of ADA is believed to stem from its strong association with rejection sensitivity.^5^, ^6^, ^7^ These findings suggest that ADA is not a rare symptom complex and is prevalent across a wide range of psychiatric disorders.

The aim of this study is to explore the phenomenological characteristics of ADA in greater detail and clarify the psychopathological nature of this frequently overlooked condition.

## SUBJECTS

Participants were recruited from a cohort of 4767 first‐time patients who visited the Akasaka Clinic of the Warakukai Medical Corporation between July 2017 and December 2022. Eligible participants were men and women aged 16 years or older who provided written informed consent after receiving a full explanation of the study's purpose. Exclusion criteria included high suicide risk (defined as a score of 10 or higher on the Psychiatric Brief Structured Interview), severe physical comorbidities, or cognitive impairments. Of the 343 individuals who met the eligibility criteria, 54 were excluded due to incomplete data, yielding a final analytical sample of 286 participants.

## METHODS

### Diagnosis of ADA

The diagnosis of ADA according to the criteria was made independently by a board‐certified psychiatrist (over 50 years of clinical experience) and licensed and certified clinical psychologists who had been working as medical secretaries for that psychiatrist for more than 1 year, based on the predefined diagnostic criteria outlined in Table 1. Cases in which the assessments of the two raters were inconsistent were excluded from analysis. The current status of ADA symptoms was evaluated using the ADA Questionnaire (Supplement 1). The diagnostic criteria and questionnaire were developed by one of the authors (H.K.), who has over 25 years of clinical experience in the management of ADA and has published more than 10 papers on this topic since 2016. The diagnostic criteria were initially developed in 2017 based on long‐term clinical observations of patients with ADA and were first reported in an English‐language journal.^4^ Following subsequent clinical experience and findings from our previous ADA studies, the criteria were revised in 2020 to the current five‐item version, which has since been reported in several English‐language publications.^2^, ^5^, ^6^, ^7^, ^8^ The ADA Questionnaire was originally developed in 2016 from the ADA Severity Scale (2008) to systematically assess the phenomenological features of ADA in routine clinical practice. It was subsequently revised and expanded to a 35‐item questionnaire in 2018 following accumulated clinical experience and discussions among the authors. Although the questionnaire has been used consistently in our clinical studies, its psychometric reliability and validity have not yet been formally evaluated.

**Table 1:** Diagnostic criteria for anxious‐depressive attack.^5^

A. Anxious‐depressive attack occurs suddenly and recurrently regardless of the situation in various psychiatric disorders. B. The following symptoms are in descending order, but symptom No. 4 is optional. 1. A sudden feeling of ominous dread, followed by a feeling of intense discomfort consisting of mixed emotions of anxious and depressive nature with or without tearfulness. A peak comes within a few seconds or less than a minute. (It seems the same as a panic attack.) 2. Intrusive rumination involving mostly negative memories, consisting mainly of recent or past adverse events or future worries, lasting from several minutes to throughout the day. It may appear as a flashback. 3. Prominent agitation, restlessness, or loneliness that occurs during rumination and is very intense and inappropriate to the ruminative content. 4. Various coping behaviors^a^ to manage intense discomfort occasionally appear.
C. Eyes filled with tears are often seen, but the other physical symptoms, such as shortness of breath and palpitations, if present, are extremely modest. D. The disorder is not due to the direct psychological effects of stress, the physiological effects of a substance, or a neurological or other medical condition. E. The disorder is not better explained by another neuropsychiatric disorder (e.g., panic disorder, major depressive disorder with anxious distress, dissociative disorders, post‐traumatic stress disorder [PTSD], and focal epilepsy).

^a^ Appx. main contents: going to bed in a huff, contacting someone, changing location, smoking, drinking alcohol, pathological shopping, binge eating, overdose, fugue, assaulting people or objects, explosive behavior, sexual derailment, self‐harm, and suicidal behavior.

### Statistical analysis

Inter‐rater diagnostic reliability for ADA was assessed using Cohen's kappa coefficient. Group comparisons were conducted using chi‐square tests and independent‐sample *t*‐tests as appropriate.

### Ethical approval

This study was conducted in accordance with the Declaration of Helsinki and was approved by the Ethics Review Board of the Medical Corporation Warakukai. Written informed consent was obtained from all participants prior to study participation. All methods were performed in accordance with the guidelines and regulations outlined in editorial and publishing policies of the journal.

Since the distinction between the terms “consciousness” and “awareness” is important in this paper, we will briefly describe how the authors use these concepts here: Although the terms consciousness and awareness are sometimes used interchangeably, they can be conceptually distinguished. Consciousness may refer to the overall state in which subjective experience is possible, whereas awareness refers to the contents of consciousness; self‐reflective awareness more specifically involves the capacity to monitor and recognize one's own mental state.^9^ Thus, an individual may remain conscious while having limited self‐reflective awareness of an ongoing mental experience.

## RESULTS

During the psychiatric interview, all patients appeared normal and responded appropriately to the interviewer's questions. The patient's behavior was appropriate for the setting.

**Figure 1:**
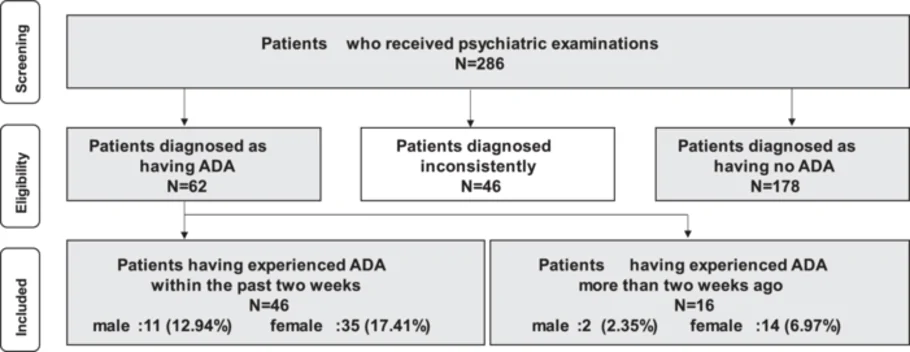
Diagram of patient selection for subjects. ADA, anxious‐depressive attack.

The diagnosis of ADA was made independently by two raters. Of 286 patients, 62 were diagnosed with ADA by both raters, while 46 showed discordant diagnoses. The Cohen's kappa coefficient was 0.61. Based on the consensus diagnoses, the lifetime frequency of ADA was 21.7%. The frequency did not significantly differ by sex: 13 out of 85 males (15.3%) and 49 out of 201 females (24.4%) were diagnosed with ADA (*χ* ^2^ = 2.39, *p* = 0.12) (Figure 1).

ADA was observed across a wide range of Axis I disorders (*n* = 13), including PD and major depressive disorder, as well as Axis II disorders (*n* = 6), including borderline personality disorder, and neurodevelopmental disorders such as autism spectrum disorder and attention‐deficit/hyperactivity disorder, totaling 21 distinct diagnostic categories (Supplement 2).

The age of ADA onset ranged from 6 to 44 years, with a median age of 20 years (males: 6–41 years, median 23; females: 9–44 years, median 19). The duration between the first and last ADA episode ranged from 62 to 86 months in males (median: 78 months) and from 42 to 415 months in females (median: 62 months).

The following results are shown in Table 2.

(1) Physical symptoms during ADA (Table 2.1)
  Physical symptoms were reported by 54.9% of patients, with 19.4% experiencing four or more types of symptoms. These findings suggest that nearly 20% of patients may have experienced PAs.
(2) Situation of ADA of the onset (Table 2.1)
  The most common time of ADA onset was between 8:00 p.m. and 4:00 a.m. (46.8%). Overall, 62.9% of patients experienced ADA during the evening to midnight hours. Approximately 70% of patients reported being alone in their rooms, doing nothing in particular, and not engaged in any specific thought activity at the time of onset. Tearfulness around the time of ADA was reported by 59.7% of participants.
(3) Mental state of ADA at onset (Table 2.1)
  About 90% of patients reported that ADA episodes occurred suddenly and unexpectedly. Emotional responses included anxiety/agitation (59.7%), followed by depression and self‐loathing (each 32.3%). Various other negative emotions were also reported, with 54.8% of participants describing the emotional intensity as “severe.”
(4) Nature of intrusive thoughts (Table 2.2)
  A total of 79.0% of patients reported intrusive thoughts with clear details during ADA episodes. The remaining 21% of patients were unable to describe them in detail. The most frequently reported number of autobiographical themes was two (42.9%), followed by one (28.6%), with only 2.0% recalling six or more themes. These memories were typically temporally distant: 32.7% dated back more than 10 years, 14.3% to 5–10 years prior, and 20.4% to 1–5 years prior. Memory clarity was generally high, with 69.4% of patients reporting almost complete recall and 30.6% reporting that they remembered most aspects of the memory but could not recall certain details. The memories were typically vivid, and 83.7% of patients rated their emotional intensity as strong. Common themes included: “I said something rude to a friend,” “I was excluded from a group,” “I was scolded by a teacher at school,” and “My mother favored my younger brother.” Six participants identified their intrusive thoughts as involving traumatic content, such as motor vehicle accidents, incidents of violence, maternal self‐injury, severe scolding, and two instances of sexual trauma. However, these memories did not consistently represent the central content of the intrusive thoughts, but were instead part of a broader set of recollections. Accordingly, the contents of these intrusive thoughts are not considered traumatic events as defined within the diagnostic framework of post‐traumatic stress disorder.
(5) Mental status during ADA (Table 2.2)
  The predominant emotions experienced at the onset of ADA were anxiety/agitation, depression, and loneliness, although a variety of other emotions were also reported. On average, each patient experienced 2.8 types of emotions. A total of 81.6% of respondents rated the intensity of these emotions as strong.
(6) Coping behaviors during ADA (Table 2.3)
  Some form of coping responses were observed in 75.8% of cases, with 22.6% exhibiting severe deviant behavior and 17.7% exhibiting mild deviant behavior. Patients who exhibited severe deviant behaviors were diagnosed with BPD.
(7) ADA status over the past 2 weeks (Table 2.3)
  The most common frequency was once or twice per week (39.1%), followed by daily episode in 32.6% of participants. The most common duration is 2–3 h (43.5%), and 23.9% of participants experience ADA lasting more than 3 h. A total of 81.6% of respondents described these emotions as strong. Additionally, 60.9% of participants reported that ADA occurred at approximately the same time on each occasion. ADA is most likely to occur during the evening through to late night hours.
(8) Recognition and interpretation of ADA (Table 2.3)

**Table 2.1:** Results about key situational, clinical, behavioral, and awareness characteristics of anxious‐depressive attack (ADA).

① Physical symptoms during ADA			
Number		*n* = 62	(100%)
	No	25	(40.3)
	1–3	22	(35.5)
	4–	12	(19.4)
	No answer	3	(4.8)
② The situation of the onset of ADA			
Time		*n* = 62	(100%)
	4–10 o'clock	5	(8.1)
	10–16 o'clock	12	(19.4)
	16–20 o'clock	10	(16.1)
	20–4 o'clock	29	(46.8)
	No answer	6	(9.7)
Place		*n* = 62	(100%)
	Own room	41	(66.1)
	Home other than own room	11	(17.7)
	Work place	4	(6.5)
	Public space	3	(4.8)
	No answer	3	(4.8)
With someone		*n* = 62	(100%)
	Alone	44	(71.0)
	With someone	15	(24.2)
	No answer	3	(4.8)
Doing something		*n* = 62	(100%)
	Doing nothing	47	(75.8)
	Yes	12	(19.4)
	No answer	3	(4.84)
Thinking of anything special just before the ADAs		*n* = 62	(100%)
	Nothing special in mind	42	(67.7)
	Yes	17	(27.4)
	No answer	3	(4.84)
Presence of lacrimation		*n* = 62	(100%)
	Some tears	37	(59.7)
	No	22	(35.5)
	No answer	3	(4.8)
③ The state of ADA at onset			
Arose suddenly and without any trigger		*n* = 62	(100%)
	Yes	55	(88.7)
	No	4	(6.5)
	No answer	3	(4.8)
Emotion type^a^		*n* = 62	
	Anxiety/agitation	37	(59.7)
	Depression	20	(32.3)
	Sadness	13	(21.0)
	Emptiness	13	(21.0)
	Self‐loathing	20	(32.3)
	Loneliness	13	(21.0)
	Hopelessness	17	(27.4)
	Angry	11	(17.7)
	Other	4	(6.5)
	No answer	3	(4.8)
Emotion strength		*n* = 62	(100%)
	Weak	4	(6.5)
	Medium	21	(33.9)
	Strong	34	(54.8)
	No answer	3	(4.8)

^a^ Multiple answers allowed; percentages based on total respondents (*n* = 62).

**Table 2.2:** Results about key situational, clinical, behavioral, and awareness characteristics of anxious‐depressive attack (ADA).

④ The nature of intrusive thought			
Presence of intrusive thoughts		*n* = 62	(100%)
	Yes	49	(79.0)
	No answer	13	(21.0)
Themes^a^, ^b^		*n* = 49	
	Partner	8	(12.9)
	Family	19	(30.6)
	Workplace‐related	13	(21.0)
	School‐related	12	(19.4)
	Others (unspecified)	26	(41.9)
Number of memories^b^		*n* = 49	(100%)
	1	14	(28.6)
	2	21	(42.9)
	3	7	(14.3)
	4	2	(4.1)
	5	3	(6.1)
	Over 6	1	(2.0)
	No answer	1	(2.0)
When^b^		*n* = 49	(100%)
	Within 3 months	9	(18.4)
	Within 1 year	6	(12.2)
	Within 5 years	10	(20.4)
	Within 10 years	7	(14.3)
	More than 10 years ago	16	(32.7)
	No answer	1	(2.0)
Strength^b^		*n* = 49	(100%)
	Weak	5	(10.2)
	Medium	3	(6.1)
	Strong	41	(83.7)
Traumatic^b^		*n* = 49	(100%)
	Yes	6	(12.2)
	No	43	(87.8)
Clarity^b^		*n* = 49	(100%)
	Almost complete memory	34	(69.4)
	Partially don't remember	15	(30.6)
⑤ Mental status during ADA			
Presence of emotional response		*n* = 62	(100%)
	Yes	49	(79.0)
	No	7	(11.3)
	No answer	6	(9.7)
Emotion type^a^, ^c^		*n* = 49	
	Anxiety/agitation	21	(33.9)
	Depression	16	(25.8)
	Sadness	19	(30.6)
	Emptiness	11	(17.7)
	Self‐loathing	19	(30.6)
	Loneliness	11	(17.7)
	Hopelessness	17	(27.4)
	Angry	13	(21.0)
	Other	6	(9.7)
Emotion strength^c^		*n* = 49	(100%)
	Weak	3	(6.1)
	Medium	6	(12.2)
	Strong	40	(81.6)

^a^ Multiple answers allowed; percentages based on total respondents (*n* = 62).

^b^ Applies to participants with intrusive thoughts.

^c^ Applies to participants with emotional responses.

**Table 2.3:** Results about key situational, clinical, behavioral, and awareness characteristics of anxious‐depressive attack (ADA).

⑥ Coping behaviors during ADA			
Presence of coping behavior		*n* = 62	(100%)
	Yes	47	(75.8)
	No	12	(19.4)
	No answer	3	(4.8)
Coping behavior type		*n* = 62	(100%)
	No action	12	(19.4)
	Dealing alone^a^	18	(29.0)
	Dealing with others^b^	4	(6.5)
	Substance intake and transfer^c^	11	(17.7)
	Aggression. Dependence, others^d^	14	(22.6)
	No answer	3	(4.8)
⑦ Current state of ADA			
Frequency		*n* = 46	(100%)
	1–2 times a week	18	(39.1)
	3–4 times a week	13	(28.3)
	Everyday	15	(32.6)
Duration		*n* = 46	(100%)
	~60 min	15	(32.6)
	60–180 min	20	(43.5)
	More than 180 min	11	(23.9)
Severity		*n* = 46	(100%)
	Weak	6	(13.0)
	Medium	12	(26.1)
	Strong	28	(60.9)
Regularity		*n* = 46	(100%)
	Same time period	28	(60.9)
	Different time period	17	(37.0)
	No answer	1	(2.2)
Time^e^		*n* = 28	
	4–10 o'clock	2	(4.3)
	10–16 o'clock	1	(2.2)
	16–20 o'clock	5	(10.9)
	20–4 o'clock	17	(37.0)
	Multiple answers	3	(6.5)
⑧ Recognition and interpretation for ADA			
General experiences		*n* = 62	(100%)
	No	28	(45.2)
	Yes	27	(43.5)
	No answer	7	(11.3)
Disease awareness		*n* = 62	(100%)
	No	21	(33.9)
	Yes	31	(50.0)
	No answer	10	(16.1)
Disclosure to others^f^		*n* = 62	
	None	18	(29.0)
	Parents	21	(33.9)
	Siblings	3	(4.8)
	Partners	5	(8.1)
	Friends	9	(14.5)
	Doctors/psychologists	7	(11.3)
	Others	3	(4.8)

^a^ Sleeps, cries, screams, paces, or uses distractions (e.g., TV) to escape ADA.

^b^ Seeks contact with family, friends, or acquaintances.

^c^ Takes usual/slightly more medication, overeats, shops, or goes out aimlessly.

^d^ Self‐harm/suicide attempts, aggression, property damage, substance abuse, or excessive spending.

^e^ Asked only to participants whose ADA occurred at the same time. Percentages are based on patients who experienced ADA within the past 2 weeks (*n* = 46).

^f^ Multiple answers allowed. Percentages are based on all respondents (*n* = 62).

A total of 43.5% of patients believed that ADA is a mental state that anyone might experience. Meanwhile, 50% regarded it as a symptom of illness. Notably, 29.0% had never disclosed their ADA experience to anyone. Among those who did disclose, parents were the most common confidants (33.9%).

## DISCUSSION

In this cross‐sectional study, we characterized ADA as a clinically meaningful state marked by the abrupt onset of intense anxiety, prolonged duration, and the frequent presence of intrusive autobiographical rumination. ADA was observed in 21.7% of psychiatric outpatients and occurred across a wide range of diagnostic categories, supporting its transdiagnostic nature. Despite the high incidence of ADA, no cases of the ADA symptom complex have yet been reported. A review of similar cases in the literature revealed the following reports.

Brewin et al.^10^ reviewed 36 studies on intrusive imagery and identified several common characteristics as follows. These images are typically recurrent, highly vivid, and emotionally distressing, often involving content related to events in the distant past. It is uncommon for more than six distinct types of intrusive images to be reported by a single individual. The content of intrusive images commonly centers on perceived failures or public humiliation. These characteristics of intrusive imagery reported by Brewin and colleagues^10^ largely correspond to those identified in the present study. However, ADA differs in several important respects. First, ADA occurs in the context of an acute anxiety episode, resulting in a more prolonged and sustained course. Second, individuals with ADA experience intense psychological distress and may engage in maladaptive or deviant coping behaviors, potentially exacerbating the course of illness. Furthermore, unlike PTSD‐related intrusive imagery, ADA is characterized by multiple intrusive thoughts that are not necessarily tied to a single major life event. Payne et al.^11^ conducted a review of seven studies investigating the frequency of intrusive thoughts in individuals with depression. They reported that 76% of adults with depression experienced intrusive thoughts, corresponding to a risk ratio of 2.94 compared to healthy controls (25.7%).

In a previous paper,^5^ we have already performed a differential diagnosis to distinguish ADA from similar psychiatric or neurological conditions. Therefore, we will only briefly list of those similar conditions here: **PA** (prominent physical symptoms); major depressive disorder with anxious distress (without paroxysmal onset), **mixed depressive and anxiety disorder** (without paroxysmal onset), **dissociative disorders** (with disruption in consciousness); **post‐traumatic stress disorder** (with a severe traumatic experience that happens once or twice in a lifetime); **focal epilepsy** (with response to antiepileptic drugs).

PA and ADA are known to alternate in the same individual, and in some cases, both can occur within a single episode.^2^, ^4^ These clinical observations suggest that PA and ADA may share a common underlying pathophysiology while manifesting as distinct phenotypes. Accordingly, both similarities and differences can be identified between the two conditions: the shared features of PA and ADA are as follows. Both are grounded in endogenous anxiety and not necessarily triggered by specific external stimuli.^9^ Both typically begin with a flush of fear and remit following the resolution of this acute state. In addition, both are observed transdiagnostically across a wide range of psychiatric disorders. In contrast, the distinguishing features can be identified. The core symptom of ADA is the emergence of intrusive thoughts, whereas PA is primarily characterized by somatic symptoms, such as palpitations and dyspnea. PA episodes typically resolve spontaneously within minutes to an hour. In contrast, ADA episodes often persist for several hours and, in cases of Borderline Personality Disorder, may last throughout the day. These differences likely reflect distinct mechanisms of anxiety processing. In PA, anxiety is externalized and discharged through somatic symptoms, allowing for relatively rapid resolution. In contrast, ADA involves a ruminative cognitive process, in which endogenous anxiety becomes linked to self‐referential themes such as regret, humiliation, or loneliness. These emotions are repeatedly reactivated through intrusive autobiographical memories, forming a self‐reinforcing cycle that prolongs the episode. At the neural level, this distinction may reflect a shift from predominantly amygdala‐driven acute fear responses in PA to sustained interactions between the amygdala and medial prefrontal–default mode network circuits underlying self‐referential processing in ADA.^8^

Patients with PA or ADA are typically able to identify both the onset and termination of their episodes and can recall the agonic state experienced during the attack. Whether experiencing a PA or an ADA, patients during an episode focus their attention on physical distress or intrusive thoughts. This state of intense immersion leads to a reduction in self‐reflective awareness or metacognition. These observations suggest that overall consciousness is preserved, but self‐reflective awareness is reduced during PA or ADA episodes. In this sense, PA and ADA are similar, although the condition is more severe in ADA. Reduced metacognition impairs an individual's ability to objectively observe their own mental processes. As negative emotions intensify, patients may become more driven toward irrational or maladaptive coping behaviors. Metacognitive dysfunction^12^ and catastrophic thinking^13^ are not rare in PD. It is reasonable to assume that this also applies to ADA. Neuroimaging findings support this conceptualization in PD. Patients with PD exhibit significantly increased activation in the left posterior cingulate cortex in response to threatening stimuli, suggesting enhanced processing of emotionally salient autobiographical memories compared to healthy controls.^14^ Furthermore, a recent resting‐state fMRI study of PD^15^ demonstrated that activity in the medial prefrontal cortex and amygdala is positively correlated with tendencies toward catastrophizing.

In the present study, 17.7% of patients with ADA exhibited mild deviant coping behaviors, while 22.6% showed severe forms, including self‐harm. Rejection sensitivity—which we consider to be a key psychopathology of ADA—may be closely related to this situation. A study involving individuals with and without a history of depression showed that rejection sensitivity (RS) predicts increased rumination.^16^ Rumination has also been associated with self‐injury and suicidal ideation.^17^ Moreover, RS itself has been identified as a significant contributor to self‐harming behavior.^18^

The present study revealed that only half of patients regarded ADA as a symptom of illness. Notably, 29.0% had never disclosed their ADA experience to anyone. Many patients perceive ADA as a common psychological experience rather than a pathological symptom. ADA is characterized by high ego‐syntonicity, meaning symptoms are deeply internalized and rarely verbalized. Unlike PA, which is marked by prominent somatic symptoms that are more readily observable as pathological, ADA tends to remain covert. This study showed that around 70% of patients said they were alone in their rooms, not doing anything in particular and not thinking about anything specific, when ADA began. It is considered that ADA episodes frequently occur during moments of mental disengagement, when the individual is not engaged in goal‐directed activity or explicit thought processes. These episodes often arise in a state of self‐absorption, in which sensory input from the external world is diminished. Consequently, patients with ADA may find it particularly difficult to observe and describe their symptoms from an objective perspective. Despite the intense suffering involved, patients rarely initiate complaints about ADA on their own. Nonetheless, certain phrases used by patients may indirectly suggest the presence of ADA. Examples include: “Suddenly, the depression came over me”; “Anxiety attacks me—I binge on sweets. Later I feel like I was a different person”; “At night, I fall into despair. I feel incredibly sad and just want to cry”; “Suddenly I get the urge to self‐harm. I don't tell my parents because I don't want to trouble them”; and “I get so irritable I can't sit still—I end up throwing things around the room and yelling at my mother. Looking back, I feel sorry for her.” Such statements are likely to prompt clinicians to become aware of the existence of ADA.

Importantly, since ADA occurs during preserved consciousness, most patients are able to recognize the onset and termination of the episode. However, ADA can last for extended periods—sometimes for half a day, an entire day, or until sleep onset. During such prolonged episodes, patients may lose awareness that they are experiencing an attack, even while thinking or acting under its influence. In some cases, an episode may begin at night, persist until sleep, and then be followed by awakening without conscious awareness of the episode's resolution. Because of its long duration, ADA is often not recognized as an episodic phenomenon, either by patients themselves or by others.

In conclusion, ADA has likely remained underrecognized in clinical psychiatry for several reasons: ① its pronounced ego‐syntonicity makes it difficult for patients to disclose; ② it resists objective observation; ③ it tends to be prolonged; and ④ in some cases, its termination is not consciously recognized. These characteristics contribute to the underdiagnosis and limited clinical recognition of ADA to date.

## LIMITATIONS

We should mention 46 cases of diagnostic discrepancy, which is an excessive number. The breakdown of these discrepancies is as follows: In 30 cases, the psychiatrist alone determined that the criteria were met. In 16 cases, this determination was made by the psychologists alone. Assuming that diagnoses made by the experienced psychiatrist are more reliable, the prevalence rates may be higher than shown here. However, this is unlikely to significantly impact the phenomenological findings.

Furthermore, we should point out that the data presented in this study were derived from interviews with outpatients and are cross‐sectional in nature. As these findings are based on self‐reported information, they may be difficult to verify objectively. In addition, potential biases should be considered when interpreting the results, including selection bias associated with a specialized outpatient setting and recall bias. Despite these limitations, the consistent phenomenological features observed across a range of psychiatric conditions suggest that ADA is a clinically meaningful construct. From a clinical perspective, identifying ADA could help clinicians recognize unarticulated internal distress and inform interventions targeting metacognitive awareness and maladaptive rumination.

## AUTHOR CONTRIBUTIONS

Hisanobu Kaiya conceived and designed the study, collected and interpreted the clinical data, and drafted the manuscript. Takeshi Otowa and Tsukasa Sasaki reviewed critically and revised the manuscript. Shota Noda contributed to data collection and performed the statistical analyses. Naomi Matsuda contributed to data collection and management. Yojiro Sakai and Fusaka Minami critically reviewed and revised the manuscript. All authors read and approved the final manuscript.

## CONFLICT OF INTEREST STATEMENT

The authors declare no conflicts of interest.

## ETHICS APPROVAL STATEMENT

The design of this study was approved by the Ethics Committee of the Medical Corporation of WARAKUKAI (Japanese Ministry of Health, Labor and Welfare Research Ethics Review Committee Number 23000093).

## PATIENT CONSENT STATEMENT

All patients in the study gave written consent for participation in the study and for the publication of their data.

## CLINICAL TRIAL REGISTRATION

N/A.

## Supporting information

Supporting File 1

## Data Availability

The data that support the findings of this study are available from the corresponding author upon reasonable request.
