# Supplementary material for: Anxious‐depressive attack—A clinical state with intrusive rumination
Source: PCN Rep. 2026 Sep 16;5(3):e70396. doi: 10.1002/pcn5.70396 (PMC13579528; doi:10.1002/pcn5.70396)
Supplement: Supplementary file 1 [file PCN5-5-e70396-s001.docx]

Supplement　1-1. ADA Questionnaire

Supplement　1-2. ADA Questionnaire

Supplement　1-3. ADA Questionnaire

Supplement 2. Clinical　diagnoses　of　patients　with/without　ADA
